# The Incomplete Puzzle of the BCL2 Proteins

**DOI:** 10.3390/cells8101176

**Published:** 2019-09-29

**Authors:** Hector Flores-Romero, Ana J. García-Sáez

**Affiliations:** Interfaculty Institute of Biochemistry, Eberhard-Karls-Universität Tübingen, 72076 Tübingen, Germany; hector_uniupv@hotmail.com

**Keywords:** BCL2 proteins, MOMP, protein membrane interactions, apoptosis, cancer therapy

## Abstract

The proteins of the BCL2 family are key players in multiple cellular processes, chief amongst them being the regulation of mitochondrial integrity and apoptotic cell death. These proteins establish an intricate interaction network that expands both the cytosol and the surface of organelles to dictate the cell fate. The complexity and unpredictability of the BCL2 interactome resides in the large number of family members and of interaction surfaces, as well as on their different behaviours in solution and in the membrane. Although our current structural knowledge of the BCL2 proteins has been proven therapeutically relevant, the precise structure of membrane-bound complexes and the regulatory effect that membrane lipids exert over these proteins remain key questions in the field. Here, we discuss the complexity of BCL2 interactome, the new insights, and the black matter in the field.

## Perspective in BCL2 Universe

The proteins of the BCL2 family are the main regulators of the intrinsic apoptotic pathway and constitute a fundamental part of tumorigenic cell dismissal and cancer treatment effectiveness [1,2]. Apoptosis effectors, or BAX-type proteins, are the most effective removers of damaged cells, while their antiapoptotic counterparts, or BCL2-type proteins, inhibit apoptotic cell death and play a role in chemotherapeutic resistance [3,4]. The opposing forces between BAX- and BCL2-type proteins are tuned by the so-called BH3-only proteins, a third subgroup of this family of proteins that promotes apoptosis by activating BAX-type proteins and/or blocking antiapoptotic proteins [5].

**Figure 1:**
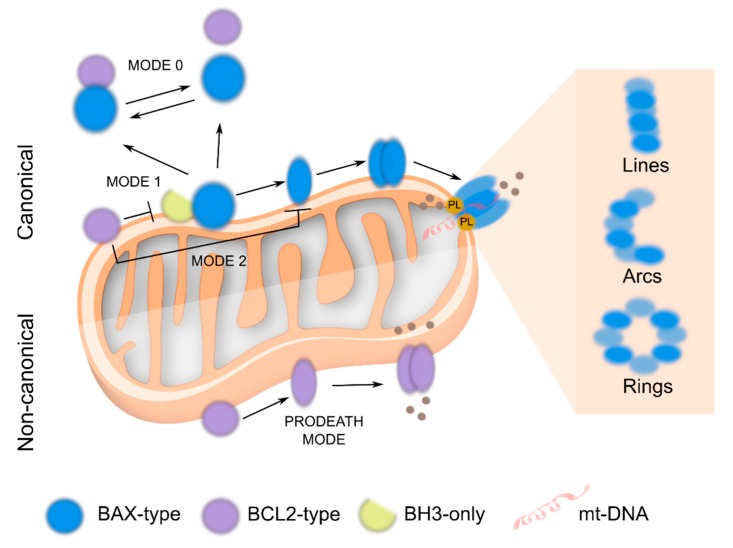
The BCL2 puzzle. *Canonical BAX/BAK activation*. Activation of BAX-type proteins at the mitochondrial outer membrane (MOM) by the BH3 only proteins induces their oligomerization, formation of supramolecular structures (lines, arcs and rings) and pore formation with the consequent release of apoptogenic factors. The apoptotic repressors, block this process by either interacting with BH3 only proteins (MODE1) or with BAX-type proteins in the membrane (MODE 2) or translocating them to the cytosol (MODE 0). *Non canonical cell death or PRODEATH MODE of BCL2-type proteins.* Under cellular stress, BCL2-type proteins can switch their antiapoptotic phenotype, directly eliciting rather than inhibiting membrane permeabilization. PL: phospholipids; grey balls: apoptogenic factors.

The proteins of the BCL2 family interact with each other by a BH3-into-groove mechanism, where the BH3 domain of one protomer binds to the hydrophobic groove of another protomer, thereby forming homo and heterodimers to control their apoptotic function. Under this premise several models have emerged, with differences in binding affinities amongst subgroups and the relevancy of the membrane environment. These models propose that antiapoptotic proteins repress apoptosis neutralizing either BH3-only activators (direct model, MODE 1) or BAX-type proteins (indirect model, MODE 2) [6,7,8,9,10,11,12,13,14]. In addition, retrotranslocation or inhibition MODE 0 postulates that BCL2-type proteins inhibit apoptosis by keeping BAX-type proteins inactive through continuous retrotranslocation from the mitochondrial surface into the cytosol [15,16,17,18,19]. These models, however, do not consider an enigmatic property shared by all BCL2-type proteins, which is their ability to promote, rather than inhibit, apoptosis under specific conditions (PRODEATH MODE) [15,20,21,22]. The complex interaction network that orchestrates these proteins’ actions is commonly termed the BCL2 interactome, which constitutes an intricate puzzle yet unresolved (Figure 1).

There are multiple reasons why, in spite of the pieces of this puzzle being defined long ago, it remains impossible to unequivocally model BCL2-mediated cell fate. First, BCL2 proteins perform their function, that is, regulating mitochondrial outer membrane (MOM) permeabilization (MOMP) to release apoptogenic factors into the cytosol and therefore induce apoptosis, when targeted to the membrane. The membrane and its constituting lipids affect the pieces of the BCL2 puzzle by modulating the affinities between the different family members [23,24] or by altering their canonical phenotype or function, for example switching their antiapoptotic nature to proapoptotic activity [15]. In addition to the membrane environment, posttranslational modifications have also been shown to modify the affinity and function of the BCL2 family members [13,25,26]. These modifications include phosphorylation, proteolytic cleavage, ubiquitination, and proteosomal degradation [14,25]. Second, despite common consensus on the importance of the BH3:groove in mediating the interaction between proteins, additional non-canonical surfaces exist that regulate the BCL2 interactome (e.g., rear binding site, N-terminal alpha helix 1 and tail anchoring domain) [27,28,29,30,31]. Third, these proteins can assemble into defined supramolecular structures that expand their role in cell death beyond cytochrome c release [32,33]. Indeed, the oligomeric apoptosis effectors BAX and BAK are able to mediate mitochondrial DNA (mtDNA) release and an immunological response [34,35,36]. Finally, some of the BCL2 puzzle’s pieces participate in other cellular puzzles, as BCL2 proteins are reported to have many other functions which are not directly related to apoptotic cell death. For example, these proteins elicit critical roles in normal cell physiology related to metabolism, mitophagy, mitochondrial dynamics and energetics, and calcium homeostasis, amongst others [37,38,39,40].

Years of extensive research efforts have shed light on important mechanistic and structural details of the BCL2 family proteins [41,42,43]. Understanding the atomic structure and interaction network of these proteins has provided fundamental opportunities for the rational design of drugs that specifically target them. These compounds, commonly known as BH3 mimetics, are molecules based on the BH3 domain of BH3-only proteins designed to interact with specific BCL2 family members [44]. BH3 mimetics exhibit enhanced lethal activity in primed cells, which contain high levels of antiapoptotic and proapoptotic effectors [44,45,46]. Chief amongst them is Venetoclax (or ABT-199); based on the BH3 only protein BAD, this compound efficiently neutralizes BCL2, thereby leading to BAX/BAK activation and to MOMP to induce apoptosis [47,48]. Although this drug has been recently approved to treat chronic lymphoid leukaemia (CLL), acute myeloid leukaemia (AML) and small lymphocytic lymphoma (SLL) [47,48,49,50,51,52,53,54], its applicability for cancer treatment is limited and chemotherapy still remains the most frequent alternative [55]. There are many possible explanations for the partial efficiency of BH3 mimetics, including cancer heterogeneity, the lack on specific BH3-mimetics optimized for the different BCL2 members governing cell death resistance in the tumor, and mutations or posttranslational modification in the BCL2 family members that alter their canonical function and structure [45,49,55]. On the other hand, these drugs were designed based on solution studies where the regulatory role of the membrane is neglected. Moreover, some BH3-only proteins like BIMs, a shorter isoform of the BH3-only protein BIM, are reported to kill mainly due to their membrane targeting, rather than due to interaction with the antiapoptotic family members [56].

**Figure 2:**
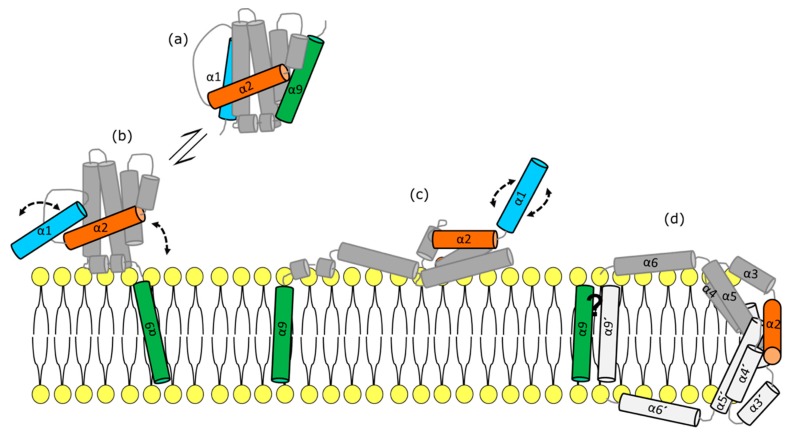
BAX/BAK structural organization during their activation process. (**a**) Protein disposition in solution. BAX is represented with nine cylinders corresponding to its nine α-helixes and based on [41]. (**b**) BAX/BAK early activation steps: including TM dislodgement and N terminal exposure (depicted in green and cyan respectively). (**c**) BAX/BAK reorganization in two different parts (dimerization and piercing domains) and BH3 domain exposure (depicted in orange). (**d**) Oligomerization and pore formation, structural representation of membrane embedded BAX/BAK in the context of toroidal pore (clamp model, based on [70]). One monomer is showed in grey (α1–9) and the other is depicted in dark grey (α1′–9′). The relative orientation of the helices 9 remains unresolved.

The exact pattern of interactions comprising the BCL2 interactome and the precise structure of the membrane-bound complexes, particularly considering the MOM environment, remains controversial [14,57,58]. Regarding the proapoptotic effectors BAX and BAK, there is solid evidence suggesting that their active conformations arrange into toroidal pores of proteo-lipidic nature and tunable size [59,60,61,62]. The structural reorganization driving BAX-type proteins from the inactive to the fully activated conformation at the MOM is considered the “holy grail” of apoptosis research [63]. These events are usually divided into: (i) early activation steps; involving TM dislodgement and N terminal exposure [64,65,66], (ii) BH3 domain exposure, which occurs due to BAX/BAK reorganization in two different parts (dimerization and piercing domains) [5,67,68,69,70], (iii) oligomerization and redistribution into apoptotic foci [33,70,71,72,73] and (iv) pore formation [59,74] (Figure 2). Importantly, these events are regulated, at least partially, by mitochondrial membranes. Although there is literature describing the topology of active BAX/BAK in the membrane [70,71,73], we still fail to understand the contribution of mitochondrial lipids in modulating their activation, oligomerization and formation of supramolecular structures at apoptotic foci during and after MOMP. BAX was recently reported to induce mtDNA release [34,36]. This renders mitochondrial apoptosis an unexpected immunological relevance, which changes the current paradigm and expands the horizons of BCL2-based therapeutics.

Concerning the antiapoptotic members of the BCL2 family, membrane lipid composition can enhance their binding affinity for the proapoptotic members [24,75] or ablate their inhibition capacity and release a hidden pore forming activity [15,20,21,76]. Although the transition of antiapoptotic BCL2 members to pro-death molecules remains poorly understood, the therapeutic potential of this phenotypic reversion should not be neglected, given that their overexpression is key in promoting resistance to chemotherapy. The membrane permeabilizing activity of BCL2-type proteins has similarities and differences to that of BAX-type proteins. Structurally, the pores formed by BCL2-type proteins are smaller, and do not require canonical BH3:groove interactions for oligomerization and pore opening [15,20,77]. Similarly to BAX-type proteins, the amphipathic alpha helix 5 of some antiapoptotic member has been reported to mediate their membrane-permeabilizing function [15,20]. Mechanistically, stimulation of the phenotypic reversion of BCL2- type proteins is diverse, including changes in pH, caspase and µcalpain cleavage, and membrane lipid composition amongst others [15,76,78,79].

Particularly, the mitochondrion-specific lipid cardiolipin (CL) has been postulated as a key regulatory element in BCL2 protein activity [15,24]. CL is implicated in many mitochondrial functions such as normal organelle ultrastructure, mitochondrial dynamics, energy metabolism and apoptosis [80,81]. Indeed, different lines of evidence indicate that the net content of CL at the MOM increases during apoptosis [82,83]. Because of its unique structural properties (e.g., two negative charges, a relatively small head group and four acyl chains), CL can form highly-curved inverted hexagonal structures [84,85,86] and laterally segregate into defined nanodomains [87,88]. These elements support the concept that CL potentially creates a unique environment for BCL2 family proteins and promotes mitochondrial membrane alterations that facilitate bilayer structure remodeling, deformation, and ultimately permeabilization. Moreover, the peroxidized isoform of CL (CLox) weakens the interaction of cytochrome c with the MIM, a process that may also contribute to ease MOMP [82,89,90].

Beyond their role in cell death, BCL2 family proteins participate in several cellular processes, including the regulation of mitochondrial dynamics [91]. New insights into the link between shape and function of mitochondria in health and disease (mitopathology) is beginning to unravel on several fronts [92]. A new connection between mitochondrial dynamics and not only cellular metabolism but also cell fate pathways may emerge from the intersection of BCL2 family proteins and mitochondrial reshaping machinery [91]. In vertebrates, the fundamental protein for mitochondrial fission is a large GTPase termed dynamin-related protein 1 (DRP1) [93]. The localization of DRP1 at constriction points to induce membrane fission is not random, but it seems to be mainly associated with MERCS (mitochondria ER contact sites) and to colocalize to apoptotic foci with the proapoptotic effectors BAX/BAK [94,95]. BCL2 proteins have been also related to Mitofusins 1 and 2 (MFN1/2), dynamin-like proteins involved in mitochondrial fusion [96]. Finally, mitochondrial cristae remodeling appears to be a fundamental step for the BAX-induced differential release of apoptotic factors at the apoptotic foci [34,36,97]. OPA1 is a key regulator of mitochondrial cristae remodeling [98], and its function appears to be regulated by the BH3 only protein tBID [99,100,101]. Thus, it is conceivable that BCL2 family proteins can elicit a direct regulatory effect over mitochondrial dynamics with diverse effects on cell death and survival.

All in all, it is striking that, after more than 30 years of BCL2 research, it is still unclear how these proteins behave specially at the membrane and how we could efficiently guide them in death and disease. Although many important mechanistic details have been uncovered during these years, the puzzle remains challenging to complete. However, we should not forget that therapeutic-regulation of apoptosis particularly by modulating the BCL2 interactome has strong therapeutic potential to combat human disease, including cancer and neurodegenerative disorders. Indeed, the design of Venetoclax based on BCL2 knowledge is the best evidence that a treatment targeting apoptotic proteins can get us closer to curing cancer. In spite of this, BCL2 regulation and drug targeting at the mitochondrial membrane remain intangible. Mitochondrial lipids regulate BCL2 proteins, both indirectly by changing the mechanical properties of the membrane, or directly by specifically modulating protein targeting, structure and function. Therefore, the understanding of BCL2 action in the membrane context appears to be compulsory, particularly in the light of two recent activities unveiled by these proteins that occur in the membrane, supramolecular organization into defined structures and mtDNA release [32,33,34,36]. In this context, it remains to be understood if the phenotypically-reverted antiapoptotic proteins share these activities with the proapoptotic effector BAX. On the other hand, function and abundance of BCL2 proteins are influenced by posttranslational modifications. As many of these modifications are governed by enzymes, their modulation could be efficiently achieved using small molecules, a suitable scenario for drug design and therapy. Moreover, there is a growing body of evidence suggesting that BCL2 proteins regulate metabolism and mitochondrial function, which are dysregulated in many disease pathologies [39,92,102]. The role of BCL2 in mitopathology, or mitochondria-related diseases, also provides new therapeutic opportunities [92,102]. Finally, as BCL2 proteins are all highly overexpressed in cancers, they represent prime candidates as antigens for anti-cancer therapy. Importantly, cellular immune responses against the BCL2 family proteins have been reported as common features in cancer patients, highlighting that these proteins are natural targets for the immune system and tumor microenvironment [103,104]. Taken together, comprehensive knowledge of the BCL2 family proteins is a highly reliable option to rationally design specific treatments that can cure on demand, alone or combined, adjusted depending on the specific BCL2 profile of patients. As BCL2 family proteins are reported to mediate many cellular processes in healthy and pathological situations, their targeting holds the potential to be unmatched.
